# Identification of a DNA Repair Gene Signature and Establishment of a Prognostic Nomogram Predicting Biochemical-Recurrence-Free Survival of Prostate Cancer

**DOI:** 10.3389/fmolb.2021.608369

**Published:** 2021-03-11

**Authors:** Gongwei Long, Wei Ouyang, Yucong Zhang, Guoliang Sun, Jiahua Gan, Zhiquan Hu, Heng Li

**Affiliations:** ^1^ Department of Urology, Tongji Hospital, Tongji Medical College, Huazhong University of Science and Technology, Wuhan, China; ^2^ Hubei Institute of Urology, Tongji Hospital, Tongji Medical College, Huazhong University of Science and Technology, Wuhan, China; ^3^ Department of Geriatrics, Tongji Hospital, Tongji Medical College, Huazhong University of Science and Technology, Wuhan, China

**Keywords:** DNA repair, prostate cancer, biochemical recurrence, nomogram, homologous recombination deficiency

## Abstract

**Background:** The incidence of prostate cancer (PCa) is high and increasing worldwide. The prognosis of PCa is relatively good, but it is important to identify the patients with a high risk of biochemical recurrence (BCR) so that additional treatment could be applied.

**Method:** Level 3 mRNA expression and clinicopathological data were obtained from The Cancer Genome Atlas (TCGA) to serve as training data. The GSE84042 dataset was used as a validation set. Univariate Cox, lasso Cox, and stepwise multivariate Cox regression were applied to identify a DNA repair gene (DRG) signature. The performance of the DRG signature was assessed based on Kaplan–Meier curve, receiver operating characteristic (ROC), and Harrell’s concordance index (C-index). Furtherly, a prognostic nomogram was established and evaluated likewise.

**Results:** A novel four DRG signature was established to predict BCR of PCa, which included POLM, NUDT15, AEN, and HELQ. The ROC and C index presented good performance in both training dataset and validation dataset. The patients were stratified by the signature into high- and low-risk groups with distinct BCR survival. Multivariate Cox analysis revealed that the DRG signature is an independent prognostic factor for PCa. Also, the DRG signature high-risk was related to a higher homologous recombination deficiency (HRD) score. The nomogram, incorporating the DRG signature and clinicopathological parameters, was able to predict the BCR with high efficiency and showed superior performance compared to models that consisted of only clinicopathological parameters.

**Conclusion:** Our study identified a DRG signature and established a prognostic nomogram, which were reliable in predicting the BCR of PCa. This model could help with individualized treatment and medical decision making.

## Introduction

Prostate cancer (PCa) is one of the most frequently diagnosed neoplasm all over the world, with an estimated 191,930 new cases and 33,330 death in 2020 in the United States (Siegel et al., 2020). The curative therapies including radical prostatectomy (RP) and radical radiation are the standard treatment for localized PCa (Mottet et al., 2017; Sanda et al., 2018), but biochemical recurrence (BCR) still occurs for approximately 20–40% of patients (Van den Broeck et al., 2019). Without secondary treatment, patients with BCR would experience clinical progression within 5–8 years, and among these, 32–45% will succumb to PCa within 15 years (Brockman et al., 2015). Thus, a marker signature that can identify patients with a high risk of BCR has great clinical value.

Genomic instability is one of the hallmarks of cancer (Hanahan and Weinberg, 2011). To maintain genome integrity, a complex DNA damage response (DDR) was developed to repair the DNA damage. Defects in DDR are associated with increased mutational load and genome instability, leading to a neoplastic transformation and proliferation (Minchom et al., 2018). The DNA repair gene (DRG) alterations were common in cancers, including ovarian cancer, breast cancer, and prostate cancer (Ali et al., 2017; Mateo et al., 2017; Mirza-Aghazadeh-Attari et al., 2019). Due to the DDR defects, cancer cells are more reliant on other repair pathways for survival, which makes DDR targeting an attractive therapeutic strategy. An important example is homologous recombination deficiency (HRD). The BRCA 1/2 are the important homologous recombination-related genes, and the germline BRCA 1/2 mutation has been confirmed as independent predictive factor for prognosis of PCa (Castro et al., 2013). The HRD is also a predictive marker for therapy with PARP inhibition (PARPi) such as Olaparib in PCa and other kinds of cancers (Kaufman et al., 2015; Mateo et al., 2015; Robson et al., 2017; Mateo et al., 2020). These issues indicated that DDR defects could be powerful prognostic factors in PCa.

In this work, we used The Cancer Genome Atlas (TCGA) and Gene Expression Omnibus (GEO) to explore the DRGs related to the prognosis of PCa and potentially to explore biomarkers of DNA repair deficiency to improve the survival of PCa patients.

## Method

### Publicly Available mRNA Data and DNA Repair Gene Sets

Data from two publicly available datasets were incorporated into our study. The level three mRNA sequencing and clinical data of TCGA-PRAD were acquired from TCGA (https://portal.gdc. cancer.gov/). The HTSeq-Counts data were downloaded and normalized with the edgeR package (Robinson et al., 2010). The GSE84042 dataset with seventy three prostate cancer samples was used as a validation dataset. The normalized mRNA expression file of GSE84042 was downloaded from GEO (http://www.ncbi.nlm.nih.gov/geo) and the relevant clinical information was retrieved from the Supplementary Material of the original literature (Fraser et al., 2017). The list of DRG was retrieved from Knijnenburg’s publication (Knijnenburg et al., 2018).

### Signature Generation and Statistical Analysis

We matched the DRG list with the TCGA-PRAD mRNA expression profile of the TCGA dataset. A univariate Cox proportional regression model was used to calculate the association between the expression of each DRG and BCR survival. Next, we used the least absolute shrinkage and selection operator (LASSO) method for variable selection in a Cox regression model to determine significant prognostic genes, and one standard error (SE) above the minimum criteria was selected. To make our model more optimized and practical, a stepwise Cox proportional hazards regression model was used. Finally, a risk score formula was calculated by taking into account the expression of optimized genes and correlation estimated Cox regression coefficients: Risk score = (exp Gene1 × coef Gene1) + (exp Gene2 × coef Gene2) + … + (exp GeneN × coef GeneN).

Patients with PCa were classified into the high- or low-risk group by ranking the given risk score. The “surv_cutpoint” function in the survminer package was used to determine the optimal cut-off value of the risk score. Kaplan-Meier analysis, the area under the (AUC) of the receiver operating characteristic (ROC) curve (using the timeROC package), and Harrell’s concordance index (C index, using the survcomp package) were used to evaluate the performance of the prognostic gene signature. The GSE84042 dataset was used for validation. The risk scores of each patient were calculated using the same formula and the optimal cut-off value was determined using the “surv_cutpoint” function.

To assess the DRG signature risk score distribution, we compared the risk scores according to different clinical status. The Mann–Whitney U test was used for comparison. Besides, the HRD scores, which was generated as a sum of genomic scar scores including the telomeric allelic imbalance (TAI) (Birkbak et al., 2012), loss of heterozygosity (LOH) (Abkevich et al., 2012), and large-scale transition (LST) (Popova et al., 2012), of TCGA dataset was retrieved from Knijnenburg’s publication (Knijnenburg et al., 2018) to assess the association between HRD score and the DRG signature status.

Gene ontology (GO) and Kyoto Encyclopedia of Genes and Genomes (KEGG) pathway analyses were performed for these with prognostic significance in univariate Cox regression using DAVID 6.8 (Huang da et al., 2009).

### Identification of Independent Prognostic Parameters for PCa

To identify independent prognostic parameters for PCa associated with the BCR-free survival and to validate the independent prognostic value of the gene signature, univariate and multivariate Cox regression analyses were performed based on the prognostic gene signature and clinical parameters, including the age at diagnosis, pathologic T stage, Gleason score, and preoperative PSA. Parameters with *p* < 0.05 based on univariate analysis were further included in the multivariate Cox regression analysis. *p* < 0.05 was considered statistically significant.

### Establishment and Validation of a Predictive Nomogram.

After testing for collinearity, independent prognostic parameters and relevant clinical parameters were included to construct a prognostic nomogram to predict 1-, 3-, and 5-year progression-free survival for PCa patients. Calibration plots of 1-, 3-, and 5-year were plotted to assess the reliability of this nomogram. Kaplan-Meier analysis, the AUC of the ROC curve (using the timeROC package), and C index (using the survcomp package) were used to evaluate the performance of the nomogram. To evaluate the efficacy of the DRG signature in improving the nomogram model performance, we also generated a clinical model with only clinical parameters using the Cox stepwise regression. The decision curve analysis (DCA) was applied to compare the performance of the nomogram model and clinical model. Integrated discrimination improvement (IDI) and net reclassification improvement (NRI) were also calculated.

### Statistical Analysis Softwares

Statistical analysis was performed using R software v4.0.2 and GraphPad Prism v8.01 (https://www.graphpad.com).

## Results

### Construction of the DRG Signature in TCGA Cohort

**FIGURE 1:**
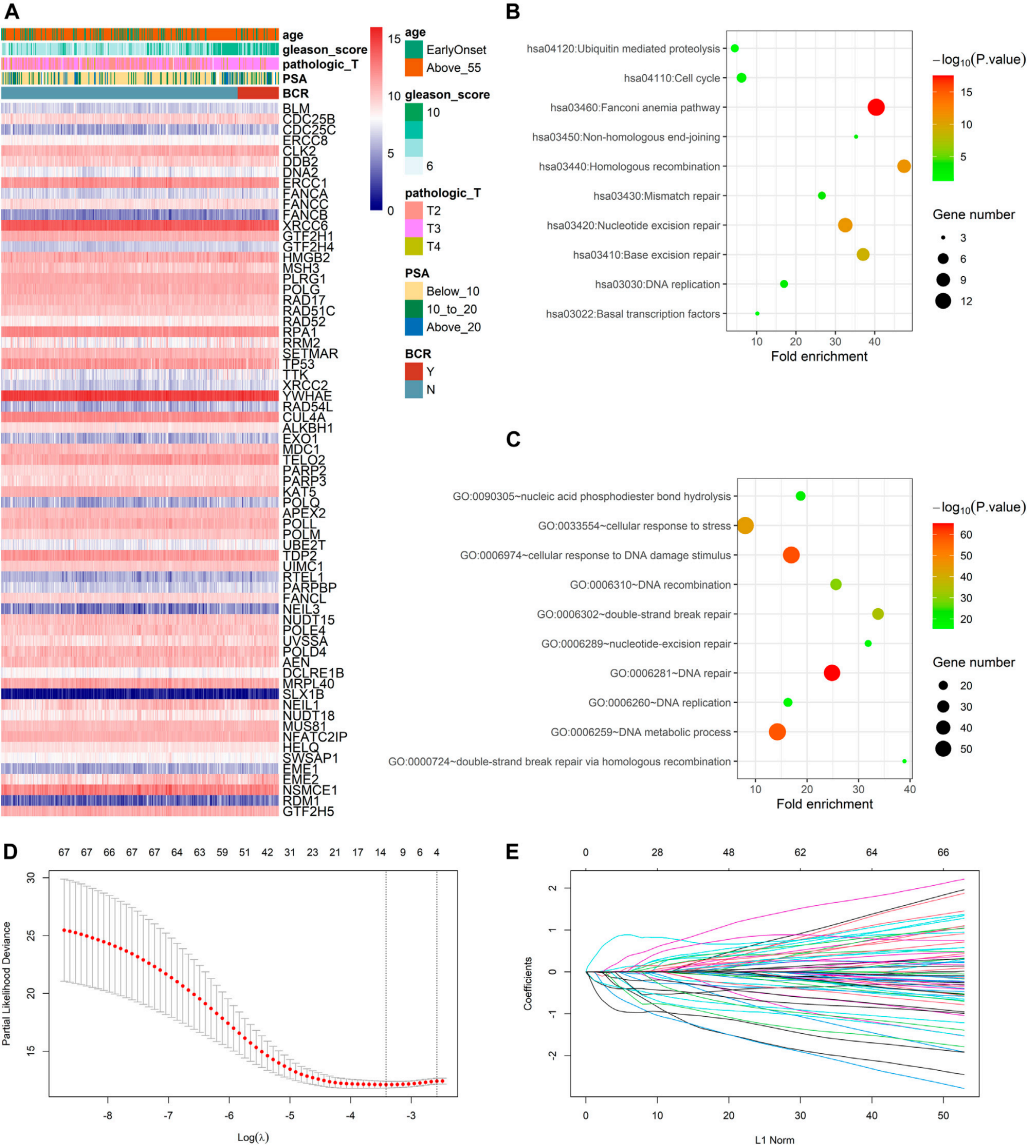
Identification of prognostic DNA repair genes in prostate cancer **(A)** Univariate Cox regression analysis identified 67 DNA repair genes significantly associated with BCR **(B)** KEGG analysis of identified genes **(C)** GO analysis of identified genes **(D)** Parameter selection in the LASSO model **(E)** LASSO coefficient profiles of the prognostic genes.

**FIGURE 2:**
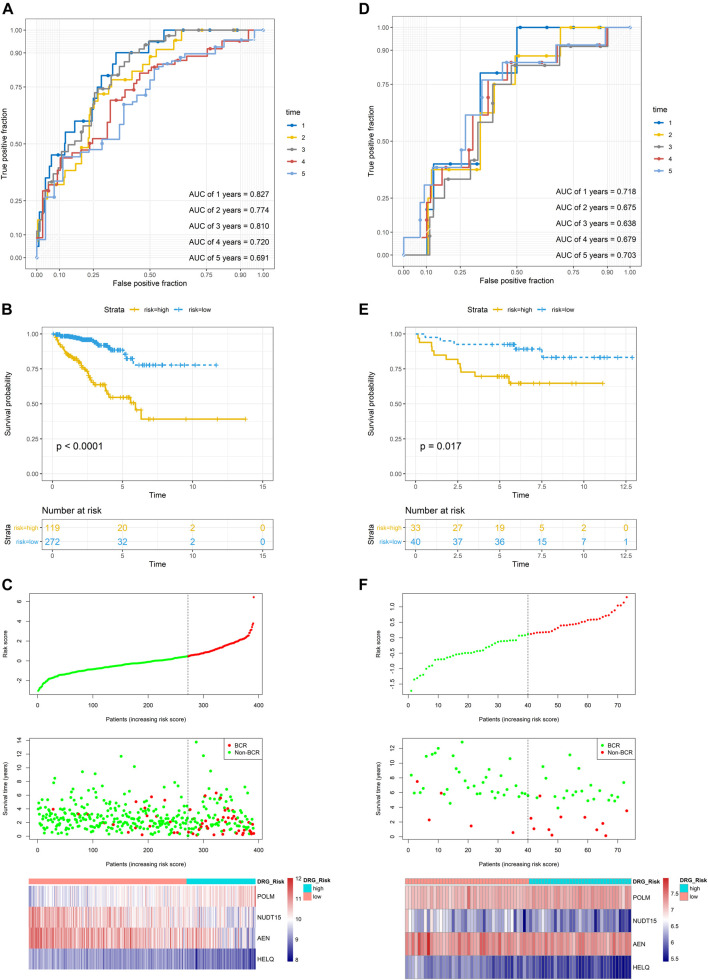
Evaluation of the prognostic performance of the DRG signature in the training dataset and validation dataset **(A)** The time-dependent ROC for 1-, 2-, 3-, 4- and 5-years BCR predictions for the DRG signature in the training dataset **(B)** The Kaplan–Meier survival curves of the DRG signature. Patients from the training dataset were stratified into two groups according to the optimal cutoff value for the risk scores **(C)** The distribution of risk score, recurrence status, and gene expression panel in the training dataset **(D)** The time-dependent ROC for 1-, 2-, 3-, 4- and 5-years BCR predictions for the DRG signature in the validation dataset **(E)** The Kaplan–Meier survival curves of the DRG signature. Patients from the validation dataset were stratified into two groups according to the optimal cutoff values for the risk scores **(F)** The distribution of risk score, recurrence status, and gene expression panel in the validation dataset.

In the TCGA dataset, three hundred and ninety one patients with the BCR survival information were selected to develop the DRG signature (Supplementary Table S1). The median (Interquartile range, IQR) follow-up duration was 2.4 (1.4–3.7) years. The univariate Cox regression analysis found that 67 DRGs were statistically significantly correlated with BCR survival (*p* < 0.05) (Supplementary Table S2). The detailed expression pattern of 67 DRG were shown in Figure 1A. KEGG and GO analyses were used to clarify the biological processes and pathways related to these significant genes (Figures 1B,C), which revealed that these genes were primarily involved in Fanconi anemia, DNA damage response, and DNA repair pathways. Next, a LASSO Cox regression model was used to calculate the most useful prognostic genes, and one SE above the minimum criteria was chosen, resulting in a model with four genes: POLM, NUDT15, AEN, and HELQ (Figures 1D,E). Additionally, a stepwise Cox proportional hazards regression model was used and it suggested that the 4-gene signature was already the optimal model. The detailed information of this signature was listed in Supplementary Table S3. Subsequently, a risk score was built: Risk Score = (0.9139 × POLM expression)−(0.7278 × NUDT15 expression)−(0.6761 × AEN expression)−(1.2567 × HELQ expression). The risk score for each patient was calculated using this formula. The ROC curve was plotted and the AUC values of different time points were calculated. Results showed that for predicting BCR-free survival in the TCGA dataset at 1st, 2nd, 3rd, 4th, and 5th year, the DRG risk score had AUC values of 0.827, 0.774, 0.810, 0.720, and 0.691 (Figure 2A). The C index of 0.777 (95% CI, 0.722–0.831) also suggested the fair performance of the DRG signature (Table 1). According to the optimal cutoff value of risk score, patients were assigned into high-risk group and low-risk group. Kaplan-Meier survival analyses showed that the rate of recurrence in the high-risk group was significantly higher than the low-risk group (Figure 2B, *p* < 0.0001). The distribution of risk score, recurrence status, and gene expression panel were illustrated in Figure 2C.

**TABLE 1:** Summary of performance of different models.

Parameters		TCGA dataset		GSE84042	
		Value and 95% CI	*p* value	Value and 95% CI	*p* value
C Index of DRG signature		0.777 (0.722–0.831)	**< 0.001**	0.634 (0.516–0.752)	**0.026**
C Index of Nomogram,		0.780 (0.722–0.838)	**< 0.001**	0.750 (0.630–0.870)	**<0.001**
C Index of Clinical model		0.711 (0.642–0.780)	**< 0.001**	0.680 (0.548–0.811)	**0.007**
C Index of Walz‘s model		0.691 (0.620–0.762)	**< 0.001**	0.678 (0.535–0.822)	**0.014**
Nomogram vs clinical model	IDI of 1 year	0.040 (0.014–0.083)	**< 0.001**	0.066 (−0.028–0.230)	0.170
	NRI of 1 year	0.511 (0.159–0.662)	**0.016**	0.374 (−0.155–0.651)	0.106
	IDI of 2 years	0.046 (0.012–0.093)	**< 0.001**	0.068 (−0.017–0.218)	0.128
	NRI of 2 years	0.452 (0.031–0.610)	**0.042**	0.335 (−0.091–0.584)	0.112
	IDI of 3 years	0.076 (0.017–0.144)	**0.010**	0.105 (−0.039–0.279)	0.138
	NRI of 3 years	0.477 (0.055–0.616)	**0.028**	0.357 (−0.094–0.576)	0.086
	IDI of 4 years	0.072 (-0.001–0.150)	0.056	0.113 (−0.031–0.280)	0.098
	NRI of 4 years	0.376 (-0.030–0.551)	0.068	0.396 (−0.005–0.618)	0.052
	IDI of 5 years	0.049 (-0.037–0.131)	0.276	0.124 (−0.012–0.295)	0.074
	NRI of 5 years	0.302 (-0.118–0.496)	0.128	0.424 (0.017–0.638)	**0.040**
Nomogram vs Walz‘s model	IDI of 1 year	0.053 (0.021–0.098)	**< 0.001**	0.057 (−0.018–0.199)	0.129
	NRI of 1 year	0.052 (0.269–0.695)	**< 0.001**	0.388 (−0.299–0.636)	0.378
	IDI of 2 years	0.057 (0.019–0.108)	**0.006**	0.068 (−0.013–0.196)	0.102
	NRI of 2 years	0.428 (0.220–0.596)	**< 0.001**	0.350 (−0.134–0.587)	0.194
	IDI of 3 years	0.110 (0.039–0.195)	**< 0.001**	0.078 (−0.042–0.193)	0.179
	NRI of 3 years	0.435 (0.208–0.579)	**0.002**	0.373 (−0.301–0.575)	0.289
	IDI of 4 years	0.139 (0.049–0.242)	**< 0.001**	0.084 (−0.012–0.235)	0.090
	NRI of 4 years	0.380 (0.157–0.539)	**0.004**	0.413 (−0.205–0.608)	0.169
	IDI of 5 years	0.121 (0.009–0.231)	**0.038**	0.087 (−0.014–0.208)	0.090
	NRI of 5 years	0.309 (0.055–0.498)	**0.024**	0.424 (−0.202–0.637)	0.209

C index, Harrell’s concordance index; DRG, DNA repair gene; IDI, Integrated discrimination improvement; NRI, Continuous net reclassification improvement.

*p* value < 0.05 was considered statistically significant and highlighted in bold.

### Validation of DRG Signature in GSE84042 Dataset

To validate the DRG signature, the GSE84042 dataset was used as a validation dataset and the relevant information was listed in Supplementary Table S4. The dataset comprised seventy three patients and the median (IQR) follow-up duration was 5.9 (5.1–7.6) years. Using the same formula, the risk scores of each patient were calculated and the cutoff value was also determined by the “surv_cutpoint” function. The AUCs for the 1-, 2-, 3-, 4-, and 5-years BCR-free survival were 0.718, 0.675, 0.638, 0.679, and 0.703, respectively (Figure 2D), and the C index was 0.634 (95% CI, 0.516–0.752) (Table 1). Kaplan-Meier survival analyses revealed that patients in the low-risk group had significantly better BCR-free survival than the high-risk group (Figure 2E, *p* = 0.017).

### Clinical Relevance of DRG Signature

**FIGURE 3:**
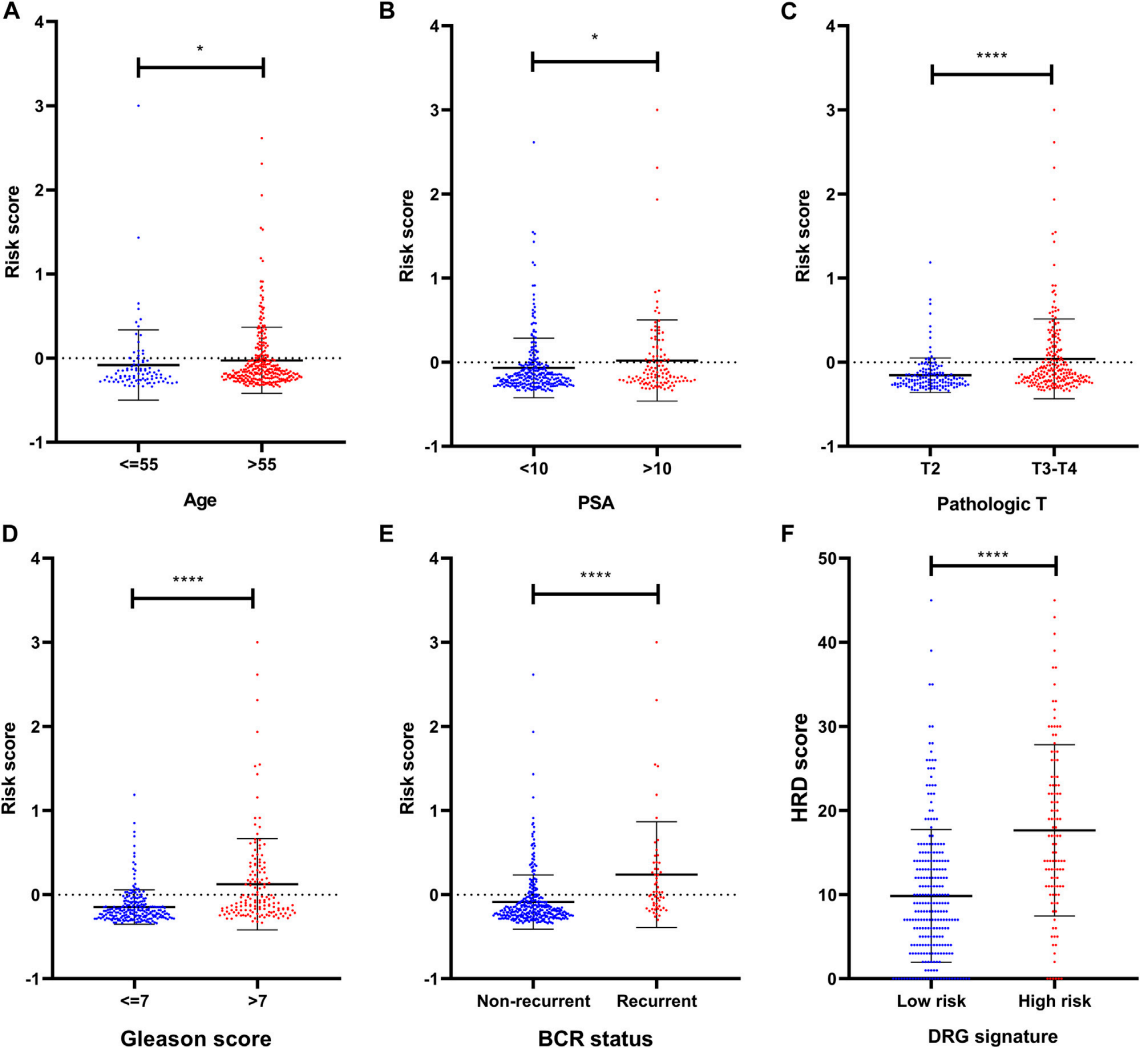
Clinical relevance of the DRG signature **(A)** The distribution of the DRG signature risk score according to different ages **(B)** The distribution of the DRG signature risk score according to different PSA **(C)** The distribution of the DRG signature risk score according to different pathologic T stage **(D)** The distribution of the DRG signature risk score according to different Gleason scores **(E)** The distribution of the DRG signature risk score according to different BCR status **(F)** The association between DRG signature and HRD score. *p < 0.05, **p < 0.01, ***p < 0.001, ****p < 0.0001. Error bars were represented as Mean with SD.

To investigate the association between clinical parameters and DRG signature, we compared the risk scores according to clinical status. Results suggested that the older age, high PSA, high pathologic T stage, and high Gleason score were related to a significantly higher DRG signature risk score (Figures 3A–D). These patients who experienced BCR also had a significantly higher risk score than those who did not recurrent (Figure 3E).

To explore the potential sensitivity to PARPi, we also compared the HRD scores in groups with different risks. The HRD status represents novel predictive biomarkers of response to PARPi (Ganguly et al., 2016) and the HRD scores could detect the HRD through its evaluation of genomic scarring based on next-generation sequencing. In our analysis, these patients who were identified as high risk by DRG signature had higher HRD scores (Figure 3F), indicating much more deficiency in homologous recombination repair in this subset of patients. In the detailed analysis, the TAI scores, LST scores, and LOH scores were all significantly higher in the DRG signature high-risk group (Supplementary Figure S1). Notably, the HRD score also presented a prognostic value in the TCGA dataset (Supplementary Figure S2).

### Identification of Independent Prognostic Parameters

We performed univariate and multivariate Cox regression analyses to evaluate the prognostic significance of the DRG signature combined with various clinical parameters (Table 2). In the TCGA cohort, the Gleason score (*p* = 0.004) and DRG signature (*p* < 0.001) were significantly correlated with BCR-free survival. Additionally, the DRG signature showed a significant prognostic value in subgroups (Supplementary Figure S3). In the GSE84042 dataset, the pathologic T stage (*p* = 0.007) and DRG signature (*p* = 0.005) were significantly correlated with BCR-free survival. Therefore, after adjustment for other clinical parameters, the DRG signature was still an independent prognostic factor for BCR-free survival in PRAD.

**TABLE 2:** Outcomes of univariate and multivariate cox regression analysis.

Variable	TCGA dataset				GSE84042			
	Univariate analysis		Multivariate analysis		Univariate analysis		Multivariate analysis	
	HR and 95% CI	*p* value	HR and 95% CI	*p* value	HR and 95% CI	*p* value	HR and 95% CI	*p* value
Age	0.995 (0.957–1.036)	0.819	NA	NA	0.980 (0.908–1.058)	0.612	NA	NA
Pathologic T (ref: T2)T3-T4	4.942 (2.233–10.940)	**< 0.001**	2.280 (0.951–5.464)	0.065	3.251 (1.124–9.408)	**0.0296**	4.467 (1.520–13.130)	**0.007**
Gleason score	2.113 (1.613–2.768)	**< 0.001**	1.541 (1.145–2.074)	**0.004**	2.208 (0.499–9.770)	0.296	NA	NA
PSA	1.021 (1.005–1.037)	**0.009**	1.008 (0.987–1.029)	0.465	1.030 (0.955–1.111)	0.442	NA	NA
DRG signature (ref: low risk) High risk	5.296 (3.013–9.310)	**< 0.001**	3.462 (1.927–6.221)	**< 0.001**	0.293 (0.101–0.850)	**0.024**	4.672 (1.580–13.810)	**0.005**

HR, Hazard ratio; DRG, DNA repair gene; NA, not applicable.

*p* value < 0.05 was considered statistically significant and highlighted in bold.In the multivariate Cox regression, only factors with a *p* value < 0.1 in the univariate analysis were included. In the TCGA dataset, the pathologic T stage, Gleason score, PSA and DRG signature were included. In the GSE84042 dataset, only pathologic T stage and DRG signature were included.

### Nomogram Establishment and Its Performance

**FIGURE 4:**
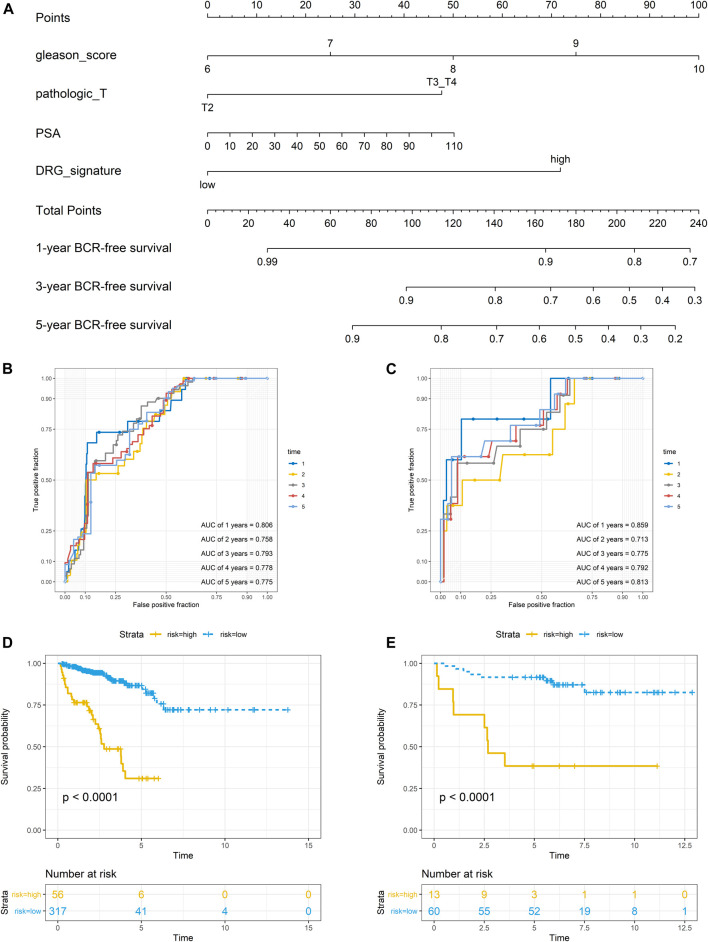
Nomogram to predict BCR-free survival probability of patients with PCa **(A)** A prognostic nomogram predicting 1-, 3-, and 5-years BCR survival of PCa **(B)** The time-dependent ROC for 1-, 2-, 3-, 4- and 5-years BCR predictions for the nomogram in the training dataset **(C)** The time-dependent ROC for 1-, 2-, 3-, 4- and 5-years BCR predictions for the nomogram in the validation dataset **(D)** The Kaplan–Meier survival curves of the nomogram. Patients from the training dataset were stratified into two groups according to the optimal cutoff values for the risk scores **(E)** The Kaplan–Meier survival curves of the nomogram. Patients from the validation dataset were stratified into two groups according to the optimal cutoff values for the risk scores.

In the TCGA dataset, three hundred and seventy three patients with complete clinical data were included to establish the prognostic nomogram. Due to the insignificant prognostic value for BCR (*p* = 0.819), the age was excluded in the nomogram establishment. The Gleason score, pathologic T stage, PSA, and DRG signature were enrolled in this model (Figure 4A). No significant collinearity was detected for all the included factors (Supplementary Table S5). The calibration plots (Supplementary Figure S4) show excellent agreement between the nomogram prediction and actual observation in terms of the 1, 3 and 5-years BCR-free survival rates in both the TCGA dataset and the GSE84042 dataset. The AUCs for the 1-, 2-, 3-, 4-, and 5-years BCR survival in TCGA dataset were 0.806, 0.758, 0.793, 0.778, and 0.775, respectively (Figure 4B) and the C index was 0.780 (95% CI, 0.722–0.838). In the GSE84042 dataset, the AUCs were 0.859, 0.713, 0.775, 0.792, and 0.813 (Figure 4C), and the C index was 0.750 (95% CI, 0.630–0.870). In the TCGA dataset, the patients were divided into high-risk and low-risk groups based on the optimal cut-off value, and the low-risk group was associated with a better prognosis (*p* < 0.0001) (Figure 4D). In the GSE84042 dataset, patients were also perfectly stratified into high-risk group and low-risk group (*p* < 0.0001) (Figure 4E).

**FIGURE 5:**
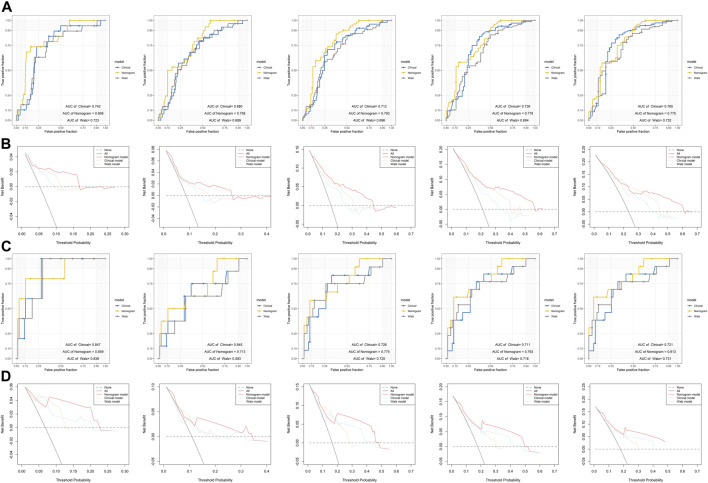
Comparison of the performance of the nomogram model, Walz’s model, and clinical model **(A)** ROC curves of the nomogram model, Walz’s model, and clinical model at 1–5 years in the training dataset **(B)** DCA curve to compare the performance of the nomogram model, Walz’s model, and clinical model at 1–5 years in the training dataset **(C)** ROC curves of the nomogram model, Walz’s model, and clinical model at 1–5 years in the validation dataset **(D)** DCA curve to compare the performance of the nomogram model, Walz’s model, and clinical model at 1–5 years in the validation dataset.

To evaluate the efficacy of the DRG signature in improving BCR prediction, a clinical model without the DRG signature was generated. We firstly input all the clinical parameters to build an initial Cox model. Then a stepwise Cox regression was applied to obtain the optimal model, which enrolled parameters including Gleason score, pathologic T stage, and PSA. Besides, we calculated the risk points of each patient using Walz’s nomogram (Walz et al., 2009). The performance of the present nomogram model, clinical model, and Walz’s model were compared. As shown in Figure 5A, the nomogram model outperformed the clinical model and Walz’s model, especially at 1–3 years. The IDI and NRI outcomes also supported the better performance of the nomogram model (Table 1). The median (IQR) follow-up duration of the TCGA dataset was 2.4 (1.4–3.7) years, and this might be the reason for the relatively insignificant improvement in the 5th year. The superior performance of the nomogram was also confirmed in the GSE84042 dataset, but the advantage was more significant in the 4th and 5th years (Figure 5C). Considering the long follow-up duration of the GSE84042 dataset, we additionally plotted the ROC curves and DCA curves at the 6th and 7th years (Supplementary Figure S5) and the improvement turned more distinct. The DCA curves suggested that the DRG signature did not bring significant net benefit for patients with very high recurrence risk in short term, but the intermedia risk population might benefit from the DRG signature (Figures 5B,D). This alerted us that the clinical parameters including pathologic stage, Gleason score, and PSA might be sufficient for very-high-risk groups, and we should select the patients to whom the DRG signature could be applied.

## Discussion

The cases of PCa is increasing worldwide, with sharp rises in incidence rates in Asia and Northern and Western Europe (Wong et al., 2016). Although the prognosis of PCa is relatively good, recurrent PCa after curative treatment may develop to progression and even metastasis. Randomized controlled trials have suggested the benefit of early androgen deprivation treatment (ADT) and radiotherapy after surgery for high-risk localized PCa (Messing et al., 2006; Gandaglia et al., 2017). The accurate prediction of prognosis will help to select patients that could benefit from further treatments. The traditional clinicopathological parameters such as TNM staging and Gleason scores can predict the prognosis of PCa, but the accuracy should be improved. Moreover, these parameters do not reflect the biological progression of PCa. Gene signatures can be measured by standardized detection systems, and dynamically describe the characteristics and progression of PCa. Additionally, these genes might represent potential therapeutic targets. Nomograms are widely used in oncology to evaluate clinical prognosis. A nomogram integrated multiple prognostic determinants including molecular biology and clinicopathological parameters, and it offers a more accurate prediction and a more intuitive view for patients. These advantages could contribute to clinical decision making and made nomogram an excellent tool for illustration of prognosis prediction (Balachandran et al., 2015).

There were many gene signatures based on different gene sets to predict the prognosis of PCa. Epigenetic alterations are frequently observed in tumors and several epigenetic biomarkers were developed including the GSTP1, APC, and RASSF1 (Trock et al., 2012; Van Neste et al., 2012; Stewart et al., 2013). Likewise, Prolaris, a gene signature consisting of thirty three cell cycle genes, was established and it was confirmed able to independently predict biochemical recurrence (Cuzick et al., 2011). Also, there were signatures comprising genes of different biological functions. The OncotypeDX Genomic Prostate Score (GPS) is based on a multi-gene assay consisting of seventeen genes related to androgen metabolism, cellular organization, proliferation, and stromal response (Klein et al., 2014). The GPS was designed to allow risk assessment for selecting candidates for active surveillance and generate valid results particularly for small tumor volumes in biopsy specimens by predicting adverse pathologic features at the time of RP, but its prognostic accuracy in predicting BCR was further confirmed (Cullen et al., 2015). The Decipher gene signature consists of a 22-gene panel and represents multiple biological pathways that are involved in aggressive PCa, including cell proliferation, cell structure, immune system modulation, cell cycle progression, and androgen signaling (Nakagawa et al., 2008). The Decipher gene signature could predict the BCR and metastasis in patients receiving postoperative radiotherapy (Den et al., 2014), and in patients following RP, it could also predict the early metastasis and even cancer-specific mortality (Erho et al., 2013; Ross et al., 2014; Klein et al., 2016; Nguyen et al., 2017; Karnes et al., 2018).

DNA repair genes play a critical role in the development of various cancer such as ovarian cancer, breast cancer, and PCa (Goodwin et al., 2013; Oktay et al., 2015; Majidinia and Yousefi, 2017). Due to the strong association between DDR defects and cancer progression, several gene signatures based on DRGs were established for cancers including ovarian cancer (Sun et al., 2019), colon cancer (Wang et al., 2020), and hepatic cancer (Li et al., 2019). In this study, we developed a DRG signature that could predict the BCR survival of PCa. Also, we built a nomogram that integrated clinicopathological parameters and the DRG signature, and the nomogram could efficiently stratify patients into a high-risk group and low-risk group. This model could provide valuable information to guide the further treatment of PCa patients who underwent RP.

Among these DDR defects, the HRD has been mostly explored. Using homologous recombination repair, a cell can efficiently perform the error-free repair of a double-strand break (DSB) in S phase. The HRD showed a double-edge property in cancer development. On the one hand, HRD resulted in genomic instability, which could a reason for the worse prognosis (Castro et al., 2013). Similar outcomes were also observed in our analysis. On the other hand, HRD is a predictor of response to specific treatment such as PARPi (Kaufman et al., 2015; Mateo et al., 2015; Robson et al., 2017; Mateo et al., 2020). The PARPi could block base excision repair, resulting in a conversion of a single strand break to a DSB. For HRD cancer cells, the accumulation of DSBs would eventually lead to cell death (D'Andrea, 2018). However, methods to identify HRD in tumors are varied and controversial (Hoppe et al., 2018). The somatic mutations in homologous recombination genes were focus biomarkers to identify HRD, and PARPi has been shown to have clinical activity in these subgroups (Mateo et al., 2015). To expand the group that suitable for PARPi treatment, a genomic-scar-based HRD score was developed, and it has been suggested as a promising predictor for response to Olaparib (Lheureux et al., 2017). In the present study, we found that the DRG signature high-risk group was related to a higher HRD score and the HRD could be a potential reason for the worse prognosis in this subset of patients. Notably, in the management of PCa, PARPi would only be considered in the castration-resistant PCa (CRPC) stage (de Bono et al., 2020; Hussain et al., 2020), and the present study was based on patients with hormone-sensitive PCa (HSPC). When the HSPC progressed to the ADT-insensitive CRPC, the genomic hallmarks also significantly changed and the proportion of HRD could also increase (van Dessel et al., 2019). In the present study, the HRD score only reflects the HRD situation at the HSPC stage, and the association between the DRG signature and HRD scores might provide information for treatment choosing when cancer progressed, but these results should be interpreted with caution.

The DRG signature consists of four genes including POLM, NUDT15, AEN, and HELQ. POLM, also known as polymerase μ (Pol μ), could promote the accuracy in the nonhomologous DNA end-joining (NHEJ), which is another solution for DSB (Waters et al., 2014). The POLM could be up-regulated in response to accumulated DSB (Mahajan et al., 2002). In our cases, the overexpression of POLM may infer the deficiency in homologous repair. NUDT15 played a role in DNA synthesis and cell cycle progression by stabilizing proliferating cell nuclear antigen (PCNA) (Yu et al., 2009). Mutations in this gene result in poor metabolism of thiopurines and are associated with thiopurine-induced early leukopenia (Yang et al., 2014). However, its role in the development of PCa was not explored. AEN (Apoptosis Enhancing Nuclease) is an autophagy-related protein-coding gene, and it is induced by p53 with various DNA damage, leading to cell apoptosis (Kawase et al., 2008; Eby et al., 2010). An association between the AEN and prognosis of hepatocellular carcinoma has been reported (Zhu et al., 2019). HELQ (Helicase POLQ-like), an ATP-dependent 3′-5′ DNA helicase, plays pivotal roles in DNA processing, including homologous recombination repair (Han et al., 2016). It has been reported to serve as an indicator of platinum-based chemoresistance for ovarian cancer (Long et al., 2018).

Besides the genomic biomarkers, several advanced examinations could also predict the prognosis of PCa. As an example, the PSMA PET/CT could predict progression-free survival in localized PCa (Roberts et al., 2020) and could even guide the use of salvage treatments such as radiotherapy (Emmett et al., 2020). However, due to the limitation of the dataset, the role of this kind of technique was unable to be adjusted in our study.

To the best of our knowledge, a prognostic model based on these five DNA repair-related genes and the associated nomogram in PCa have not been reported. A DRG signature in PCa has been previously reported to predict BCR-free, metastasis-free, and overall survival, but it is based on a profile of nine DDR pathways using seventeen gene sets for GSEA (Gene Set Enrichment Analysis) (Evans et al., 2016). Our gene signature is based on the expression of four genes. Therefore it is economical and clinically practicable to be used. Our nomogram combined with DRG signature and clinicopathological parameters presented an excellent performance in prognosis prediction. It could provide a straightforward and convenient graphical scoring system and help clinical decision making.

Our current study has some limitations. First, the training set was from the TCGA database and GSE84042 was served as the validation dataset. The majority of these patients are from North America, and thus, the expanding of our results to other ethnicities should be with caution. Second, the DCA analysis suggested that the signature did not bring significant net benefit for patients with very high risk and the signature might be more meaningful for patients who were thought moderate or low risk with traditional tools. Third, the salvage treatments could influence the BCR, and predictors such as PSMA PET could also prognosticate the BCR after salvage therapies in these patients with a rising PSA after RP (Emmett et al., 2020). While in our study, due to the lack of data, the prognostic value of the signature on patients after salvage therapies require further ascertainment. Besides, we identified four genes to construct a gene signature based on the mRNA sequencing data, but the protein expression of these genes and the underlying mechanism require further investigation. Last, the establishing and validation of this model were all conducted with publicly available data, and it needs to be further validated in original external datasets.

## Conclusion

In conclusion, our study profiled DNA repair genes that are significantly related to the prognosis of PCa. The combination of these biomarkers may serve as a signature to stratify PCa patients into low-risk and high-risk groups for assessing BCR survival. We also constructed a nomogram based on clinical parameters and the DRG signature to predict the BCR, which could be helpful for precise and personalized treatment.

## Data Availability

Publicly available datasets were analyzed in this study. This data can be found in The Cancer Genome Atlas (TCGA) and Gene Expression Omnibus (GEO) (GSE84042).
