# Supplementary figures and images for: Identification of a DNA Repair Gene Signature and Establishment of a Prognostic Nomogram Predicting Biochemical-Recurrence-Free Survival of Prostate Cancer
Source: Front Mol Biosci. 2021 Mar 11;8:608369. doi: 10.3389/fmolb.2021.608369 (PMC7991107; doi:10.3389/fmolb.2021.608369)

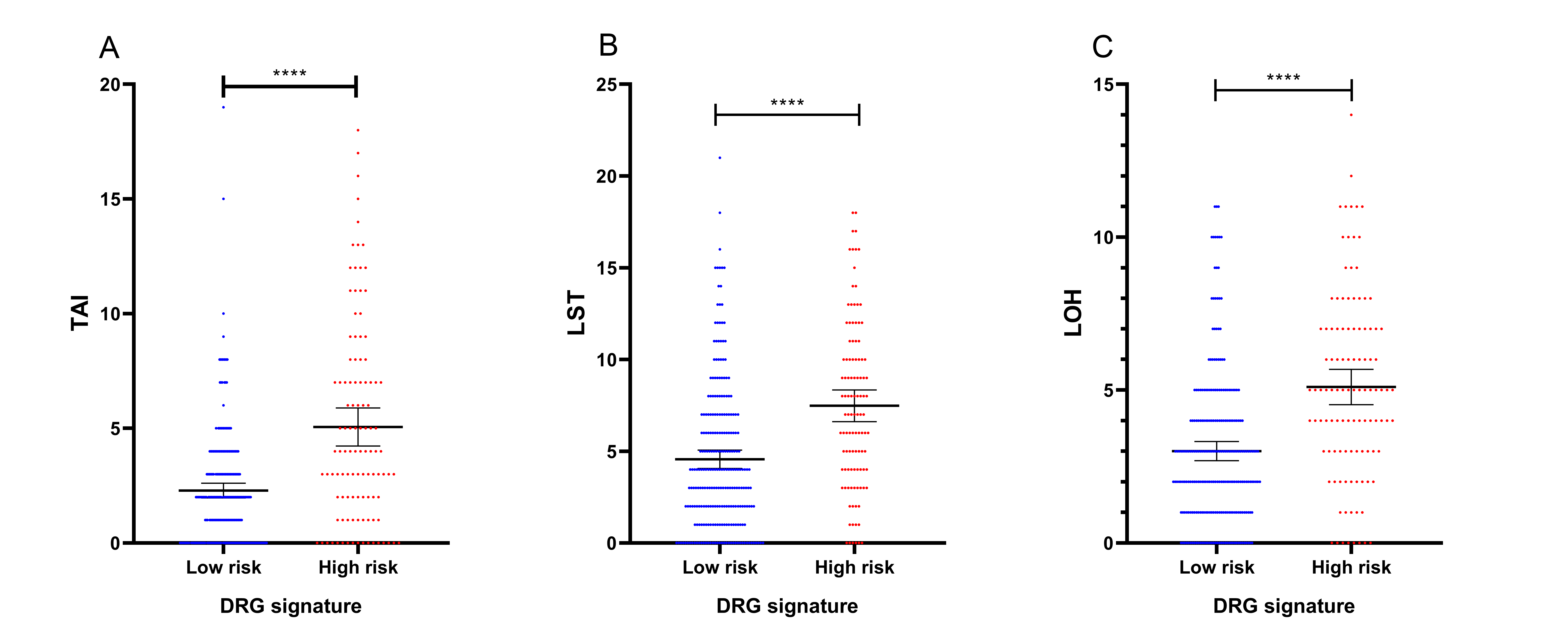

Supplement: Supplementary file 1 [file image1.tif]

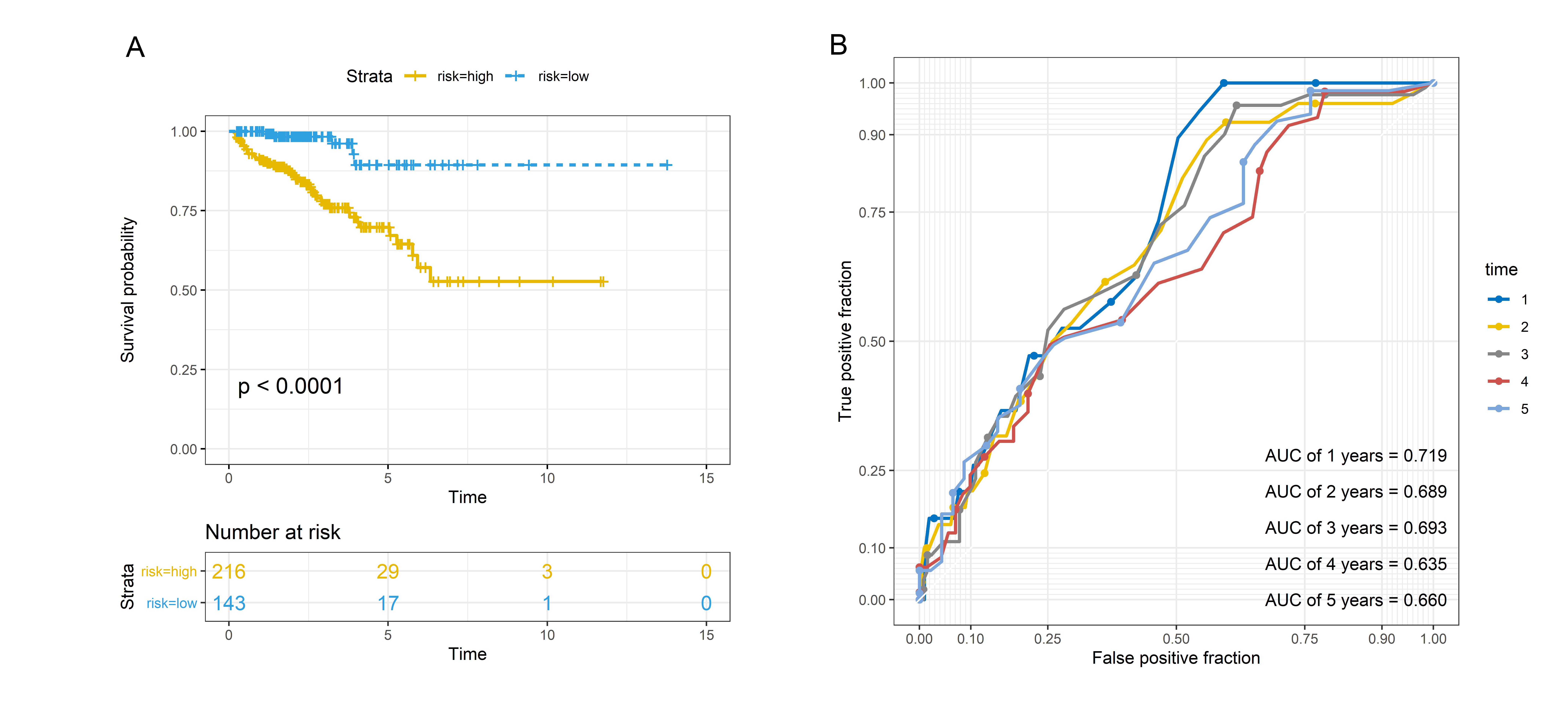

Supplement: Supplementary file 2 [file image2.tif]

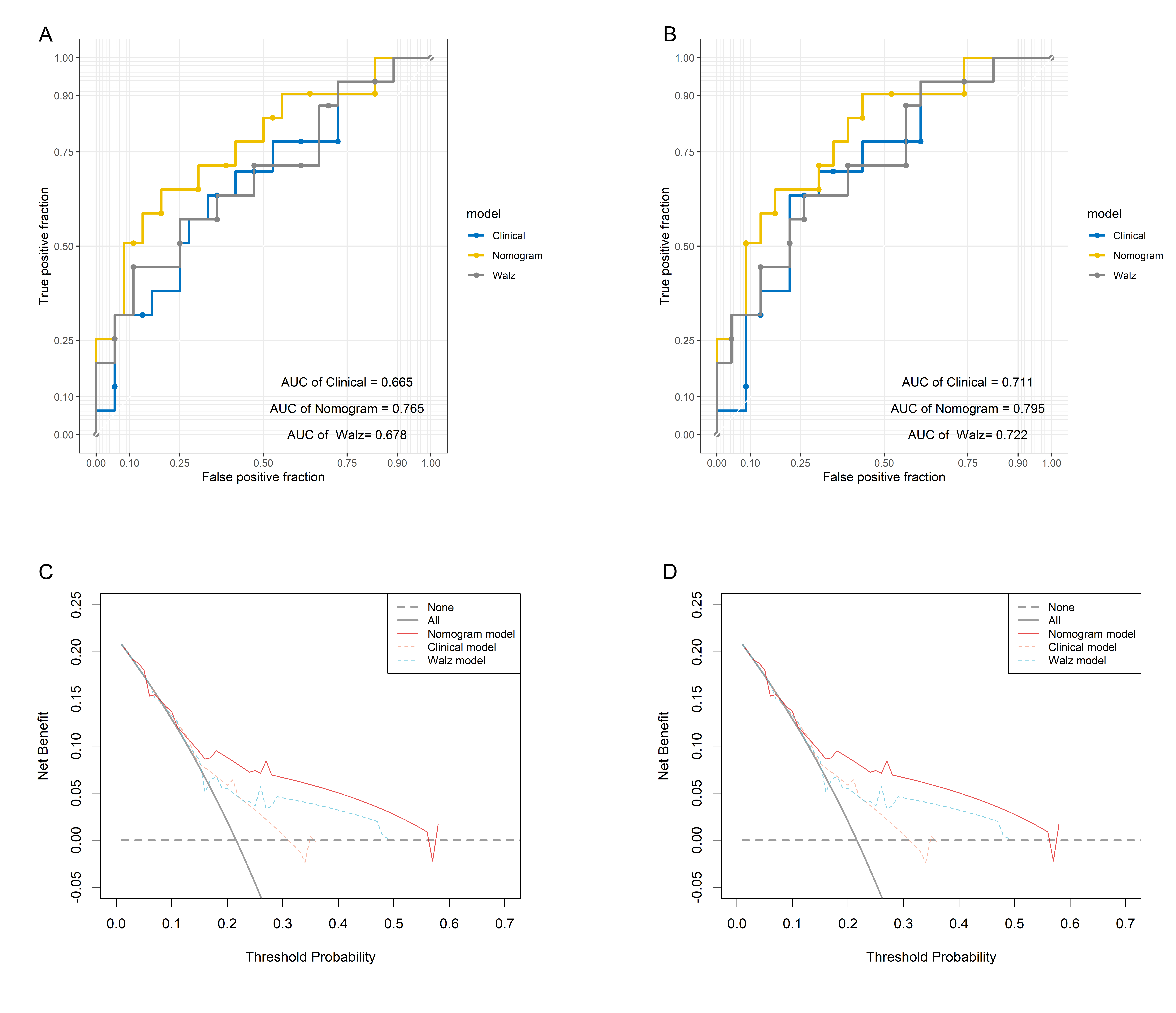

Supplement: Supplementary file 5 [file image5.tif]
