# Supplementary material for: Identification of a DNA Repair Gene Signature and Establishment of a Prognostic Nomogram Predicting Biochemical-Recurrence-Free Survival of Prostate Cancer
Source: Front Mol Biosci. 2021 Mar 11;8:608369. doi: 10.3389/fmolb.2021.608369 (PMC7991107; doi:10.3389/fmolb.2021.608369)
Supplement: Supplementary file 10 [file table5.docx]

Supplement Table 5. Collinearity analysis of included parameters in the nomograms.

|  | Collinearity analysis | |
| --- | --- | --- |
|  | Tolerance | VIF |
| Pathologic T | 0.745 | 1.343 |
| Gleason score | 0.725 | 1.380 |
| PSA | 0.913 | 1.095 |
| DRG Signature | 0.882 | 1.134 |
| Tolerance <0.1 or VIF >10 was considered collinearity positive. | | |
